# Inhibition of gap junctional intercellular communication by an anti-migraine agent, flunarizine

**DOI:** 10.1371/journal.pone.0222326

**Published:** 2019-09-12

**Authors:** Joo Hye Yeo, Eun Ju Choi, Jinu Lee

**Affiliations:** College of Pharmacy, Yonsei Institute of Pharmaceutical Sciences, Yonsei University, Songdogwahak-ro, Yeonsu-gu, Korea; Emory University School of Medicine, UNITED STATES

## Abstract

Gap junctions (GJs), which consist of proteins called connexins, are intercellular channels that allow the passage of ions, second messengers, and small molecules. GJs and connexins are considered as emerging therapeutic targets for various diseases. Previously, we screened numerous compounds using our recently developed iodide yellow fluorescent protein gap junctional intercellular communication (I-YFP GJIC) assay and found that flunarizine (FNZ), used for migraine prophylaxis and as an add-on therapy for epilepsy, inhibits GJIC in LN215 human glioma cells. In this study, we confirmed that FNZ inhibits GJIC using the I-YFP GJIC assay. We demonstrated that FNZ inhibits GJ activities via a mechanism that is independent of calcium channels and dopaminergic D_2_, histaminergic H_1_, or 5-HT receptors. In addition, we showed that FNZ significantly increases connexin 43 (Cx43) phosphorylation on the cell surface, but does not alter the total amount of Cx43. The beneficial effects of FNZ on migraines and epilepsy might be related to GJ inhibition.

## Introduction

Gap junctions (GJs) mediate cell-to-cell communication, known as gap junctional intercellular communication (GJIC), which enables the exchange of small molecules (< 1 kDa), including ions, metabolites, and nutrients, between the cytoplasm of adjacent cells. Six connexins constitute a connexon, which is joined to that of an adjacent cell to form a GJ [1]. GJs or connexins play crucial roles in the development, growth control, and homeostasis of tissues and organs, as well as the pathophysiology of various diseases including cardiovascular diseases, such as hypertrophic cardiomyopathy, heart failure, and myocardial infarction [2];[3]; particular subtypes of epilepsy [4]; migraine with aura [5]; non-neoplastic liver diseases [6]; wound healing [7]; glaucoma [8]; non-syndromic deafness [9]; X-linked Charcot-Marie Tooth disease [10]; and oculodentodigital dysplasia (ODDD) [11];[12]. In addition, GJs and connexins have been used for toxicological assessment of carcinogens, such as polycyclic aromatic hydrocarbons, that block GJs [13];[14]. Several reports have suggested that disrupted GJIC is associated with nongenotoxic carcinogenesis [15];[16]. Therefore, there is a growing interest in developing new pharmaceuticals that can modulate GJs.

Recently, we developed a cell-based high-throughput screening (HTS)-compatible iodide yellow fluorescent protein gap junctional intercellular communication (I-YFP GJIC) assay. This I-YFP GJIC assay utilizes acceptor and donor cells that express YFP^QL^, an iodide-sensitive yellow fluorescent protein variant, and SLC26A4, an iodide transporter, respectively. When iodides are added to a co-culture of acceptor and donor cells, they solely enter the donor cells via SLC26A4 and migrate to the adjoining acceptor cells via the GJs. The iodides that enter the acceptor cells quench the YFP fluorescence of the acceptor cells. Thus, the YFP fluorescence quenching rates reflect GJ activities [17][18]. We screened numerous compounds, including Food and Drug Administration approved drugs, using the I-YFP GJIC assay and identified flunarizine (FNZ) as a GJIC inhibitor.

FNZ is a versatile drug used for various pathological conditions because of its various pharmacological activities; it not only blocks calcium entry [19], but also inhibits the function of dopamine D_2_ [20], histamine H_1_ [21], and 5-HT receptors [22]. FNZ is useful in preventing migraine attacks and is also used as an add-on treatment in drug-resistant epilepsy patients, occlusive peripheral vascular disease, and central and peripheral vertigo [19]. A recent cohort study of migraine patients in the UK demonstrated that FNZ is generally effective for chronic migraine, thus encouraging the use of FNZ for migraines [23]. In this report, we demonstrate FNZ-induced GJIC inhibition in LN215 human glioma cells, as well as additional data suggesting its mechanism of action.

## Materials and methods

### Chemicals

FNZ and 5-HT were purchased from Sigma-Aldrich (St. Louis, MO, USA). Histamine and dopamine were provided by Tokyo Chemical Industry (Tokyo, Japan). The calcium channel blockers, D_2_ antagonists, H_1_ blockers, and 5-HT antagonists used in this study were obtained from Spectrum collections (MicroSource Discovery Systems, New Milford, CT, USA).

### Cell culture

Human glioma cells LN215 (a kind gift from Dr. Erwin G. Van Meir), LN215-YFP, and LN215-SLC26A4 [17] were grown in Dulbecco's Modified Eagle's medium (DMEM, Sigma-Aldrich) supplemented with 100 IU/mL penicillin, 100 μg/mL streptomycin, and 10% fetal bovine serum (FBS). FRT-Cx43 [24] cells were cultured in a 1:1 mixture of DMEM and Ham’s F-12 medium supplemented with 100 IU/mL penicillin, 100 μg/mL streptomycin, and 10% FBS. Cells were maintained in a 5% CO_2_/95% air and humidified environment at 37°C.

### Gap-fluorescence recovery after photobleaching (FRAP) assay

FRT-Cx43 cells [24] were plated on a 35-mm glass-bottomed dish coated with 2% gelatin (Sigma-Aldrich) and grown to 80% confluence. Cells were treated with vehicle or drugs diluted in C-solution (10 mM HEPES [pH 7.4], 140 mM NaCl, 10 mM glucose, 5 mM KCl, 1 mM MgCl_2_, and 1 mM CaCl_2_), as indicated. Next, 10 μM calcein-AM (Sigma-Aldrich) was added to the cells followed by a further 10 min incubation. The cells were washed twice with C-solution and incubated with the same C-solution containing vehicle or drug as in the previous treatment. Cells surrounded by more than five adjoining cells were selected using an LSM 710 confocal microscope (Zeiss, Jena, Germany) and then photo-bleached for 50 s with a maximal laser. Fluorescence images were taken 50 s prior to (-50 s) and immediately after (0 s) bleaching and then at 10 s intervals for 170 s. The percentage of fluorescence recovery was calculated as follows: % fluorescence recovery = (Ft—F_0_) / (F-50—F_0_) × 100, where F_t_ represents the fluorescence at any time point, F_-50_, the fluorescence recorded 50 s prior to photobleaching, and F_0_, the fluorescence immediately after photobleaching.

### I-YFP GJIC assay

The I-YFP GJIC assay was performed as previously described [17], with minor modifications. Briefly, a 1:4 mixture of LN215-YFP and LN215-SLC26A4 cells was plated on a 96-well plate at a density of 20,000 cells/well and incubated for 24 h. Culture media were aspirated and cells were washed twice with 200 μL of C-solution. Next, the cells were treated with vehicle or chemicals diluted in 100 μL C-solution and further incubated for the indicated period. The 96-well plate containing the cells was placed into a POLARstar microplate reader (BMG Labtech, Ortenberg, Germany). An equal volume of I-solution (10 mM HEPES [pH 7.4], 140 mM NaI, 10 mM glucose, 5 mM KCl, 1 mM MgCl_2_, and 1 mM CaCl_2_) was injected into each well at 1 s after each measurement was started at a rate of 135 μL/s using the machine-equipped automated injector. Fluorescence was measured for 20 s at 0.4 s intervals in kinetic mode using a 485 nm excitation/520 nm emission filter. The percentage (%) of YFP quenching and GJIC activity were calculated as follows:

$$YFP\;quenching\;(\%)=\left(1-\frac{YFP\;Fluorescence}{YFP\;Fluorescence\;at\;2\;s}\right)\times 100$$ (1)

$$GJIC\;activity\;(\%)=\frac{\%YFP\;quenching\;at\;20\;s}{\%YFP\;quenching\;at\;20\;s\;of\;the\;control\;group}\times 100$$ (2)

### Analysis of Cx43 on the cell surface

In situ biotinylation was conducted to analyze Cx43 located on the plasma membrane by immunoblotting. One day prior to biotinylation, LN215 cells were seeded on 100-mm plates to reach 100% confluence in 24 h and then treated with chemicals as indicated. Next, cells were placed on ice and washed three times with cold phosphate buffered saline (PBS) containing 100 mg/L CaCl_2_ and 100 mg/L MgCl_2_ (PBS-C/M). Cell surface proteins were biotinylated by adding 4 mL of 0.5 mg/mL Sulfo-NHS-Biotin (Thermo Fisher Scientific, Rockford, IL, USA) in PBS-C/M, followed by incubation with gentle rocking for 30 min. Following a wash with ice-cold PBS-C/M, 4 mL of 100 mM glycine in PBS-C/M were added to the cells for 20 min with gentle rocking to quench the remaining Sulfo-NHS-Biotin and the cells were then washed three times. The cells were lysed with cold PBS-Triton lysis buffer containing 0.6× PBS, 1% Triton X-100, 1× cOmplete Protease Inhibitor Cocktail (Roche, Basel, Switzerland) with ethylenediaminetetraacetic acid (EDTA), and 1× Halt Phosphatase Inhibitor Cocktail (Thermo Fisher Scientific). The lysates were clarified by centrifugation at 15,000 × *g* at 4°C for 10 min. Protein concentration was measured using a bicinchoninic acid (BCA) assay. To collect biotinylated proteins, 1 mg of protein was incubated with 20 μL of NeutrAvidin agarose resin (Thermo Fisher Scientific) at 4°C with gentle rotating overnight. The agarose resin was pelleted by centrifugation at 15,000 × *g* for 30 s and then washed five times with lysis buffer. Biotinylated proteins for sodium dodecyl sulphate polyacrylamide gel electrophoresis (SDS-PAGE) were eluted by incubating the pelleted resin in 50 μL of 2× Laemmli sample buffer at 37°C for 10 min.

The biotinylated protein samples extracted from 1 mg of protein lysate were separated by 8% SDS-PAGE and transferred onto nitrocellulose membranes (Whatman, Dassel, Germany) for immunoblotting. The membranes were blocked with 5% bovine serum albumin (BSA) in PBS containing 0.05% Tween 20 (PBST) for Na^+^-K^+^ ATPase, or with 5% skim milk in PBST for Cx43 and actin. Primary antibodies (anti-Na^+^-K^+^ ATPase Ab [ab7671; Abcam, Cambridge, UK], anti-Cx43 Ab [C13720; BD BioSciences, San Diego, CA, USA], and anti-actin Ab [sc-1651; Santa Cruz Biotechnology, Santa Cruz, CA, USA]) were used at a 1:1,000 dilution in the corresponding blocking solutions. The secondary anti-Mouse Ab or anti-Goat Ab conjugated with HRP (PI2000; Vector Laboratories, Burlingame, CA, USA) was used at a 1:5,000 dilution in PBST + 5% BSA or skim milk. Immunoblot images generated with enhanced chemiluminescence (ECL) solution (Thermo Fisher Scientific) were captured using a Fusion Solo 4M (Vilber Lourmat, Eberhardzell, Germany). The band intensities were analyzed using ImageJ software. The degree of phosphorylation was calculated based on the ratio of the intensity of the phosphorylated bands (P1 and P2) relative to that of the non-phosphorylated bands (P0). The total amount of Cx43 on the membrane was calculated based on the intensities of the total Cx43 bands (P0 + P1 + P2) divided by those of the Na^+^-K^+^ ATPase bands. Minimal cytosolic protein contamination in the cell surface samples was confirmed by the absence of actin.

### Assessing the change in [Ca^2+^]_in_ following FNZ treatment

LN215 cells were cultured in 96-well plates to reach 90% confluence. The Fluo4 NW Calcium Assay Kit (F36206, Thermo Fisher Scientific) was used to monitor [Ca^2+^]_in_ according to the manufacturer’s instructions. Growth media were removed and cells were incubated with 100 μL of the dye loading solution for 45 min at 37°C. Next, the cells were treated with vehicle or 50 μM FNZ and Fluo4 fluorescence was measured prior to and every 30 min after treatment with vehicle or FNZ for 240 min using a POLARstar microplate reader (BMG Labtech). As the Fluo4 dye loaded in the cells is released into culture medium at a millimolar Ca^2+^ concentration, Fluo4 fluorescence distinctly increases following 4 h of incubation. Thus, the Fluo4 fluorescence of FNZ-treated cells was normalized to that of vehicle-treated cells for each time point. To assess the rapid effect of FNZ on [Ca^2+^]_in_, the 96-well plate with cells was placed into a POLARstar microplate reader (BMG Labtech) after dye loading, as described above. Vehicle, 50 μM FNZ, or vehicle together with 100 μM ATP were added to each well using the automatic injector of the microplate reader and the Fluo4 fluorescence of each well was measured every 0.5 s for 100 s. The Fluo4 fluorescence of each group measured after the injection was normalized to that of the corresponding group at the time of injection initiation (0 s) and plotted as graphs.

### Statistical analysis

Replicate experiments were conducted at least three times and data are expressed as mean ± standard deviation (SD).

Statistical significance was assessed by Student’s *t*-test using SSPS software.

## Results

### GJIC inhibition by FNZ

**Fig 1:**
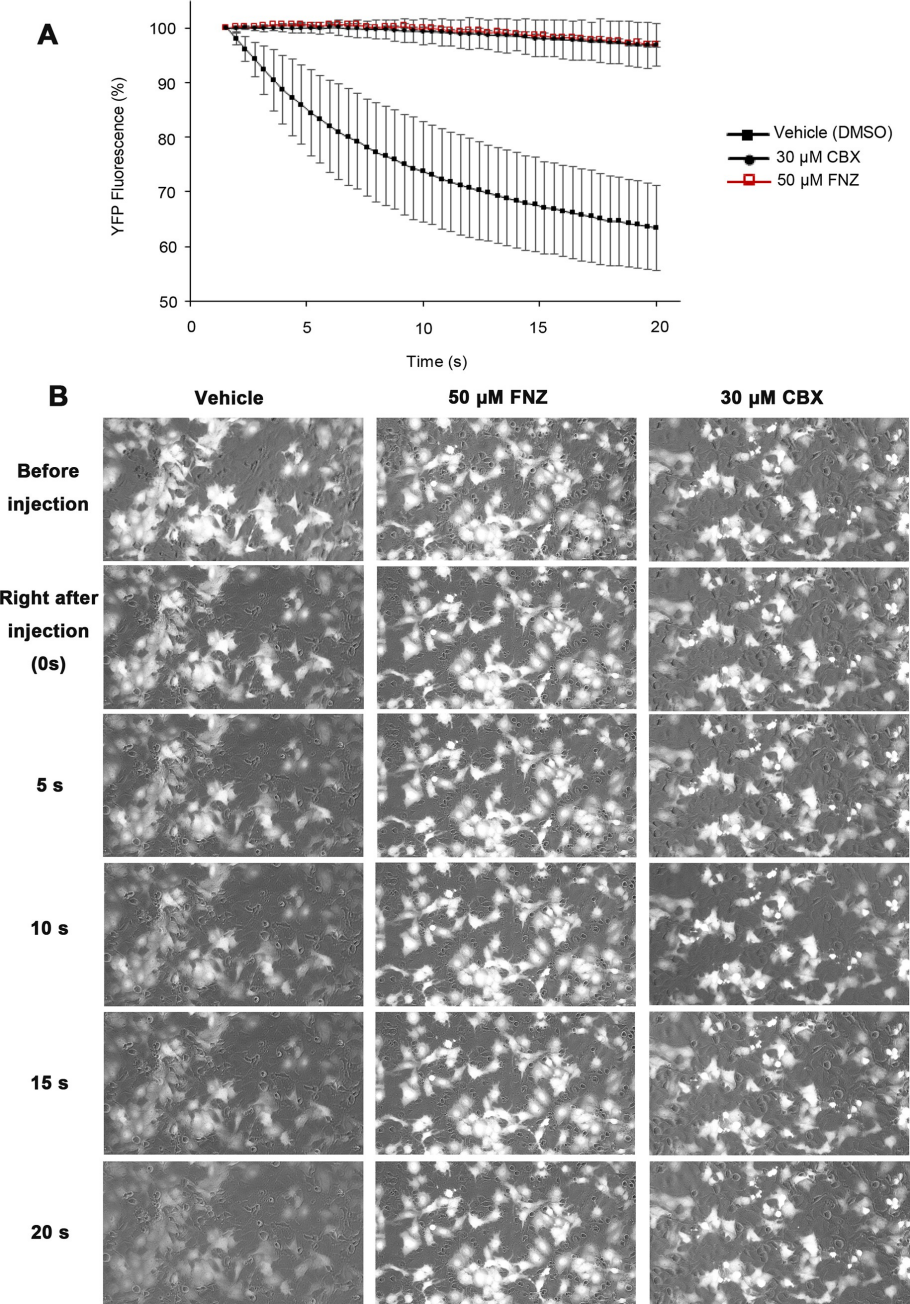
FNZ-induced inhibition of GJIC shown using the I-YFP GJIC assay. (**A**) I-YFP GJIC assay in LN215 cells. A 1:4 mixture of LN215-YFP^QL^ and LN215-SLC26A4 cells was plated on 96-well plates and cultured for 24 h. The cells were treated with vehicle, 50 μM FNZ for 4 h, or 30 μM CBX for 10 min prior to the I-YFP GJIC assay. The percentage of YFP fluorescence was plotted against time. The FNZ and CBX traces overlap. Data are presented as the mean ± SD (n = 4). (**B**) Original representative images of Fig 1A. The images were taken before, right after, and every 5 s for 20 s after iodide injection.

To assess the effect of FNZ on GJIC activity, we conducted an I-YFP GJIC assay in LN215 cells. GJIC inhibition was observed in cells treated with 50 μM FNZ for 4 h or 30 μM carbenoxolone (CBX), a well-known GJ blocker (used as the positive control), for 10 min, but not in the vehicle-treated control group (Fig 1A). The percentage of YFP fluorescence in vehicle-treated cells was quenched by 27.4% (63.4 ± 7.7% of the initial value), whereas that in FNZ- or CBX-treated cells was only quenched by ~3% (97.0 ± 0.6 or 96.8 ± 3.9% of the initial value, respectively) after 20 s (Fig 1A). Original representative images of cells in Fig 1A at different time points with different treatments are presented in Fig 1B. Since compounds that inhibit SLC26A can exhibit artifactual GJIC inhibition, we examined whether FNZ inhibits SLC26A4. The iodide uptake via SLC26A4 was not inhibited by FNZ (S1 Fig).

**Fig 2:**
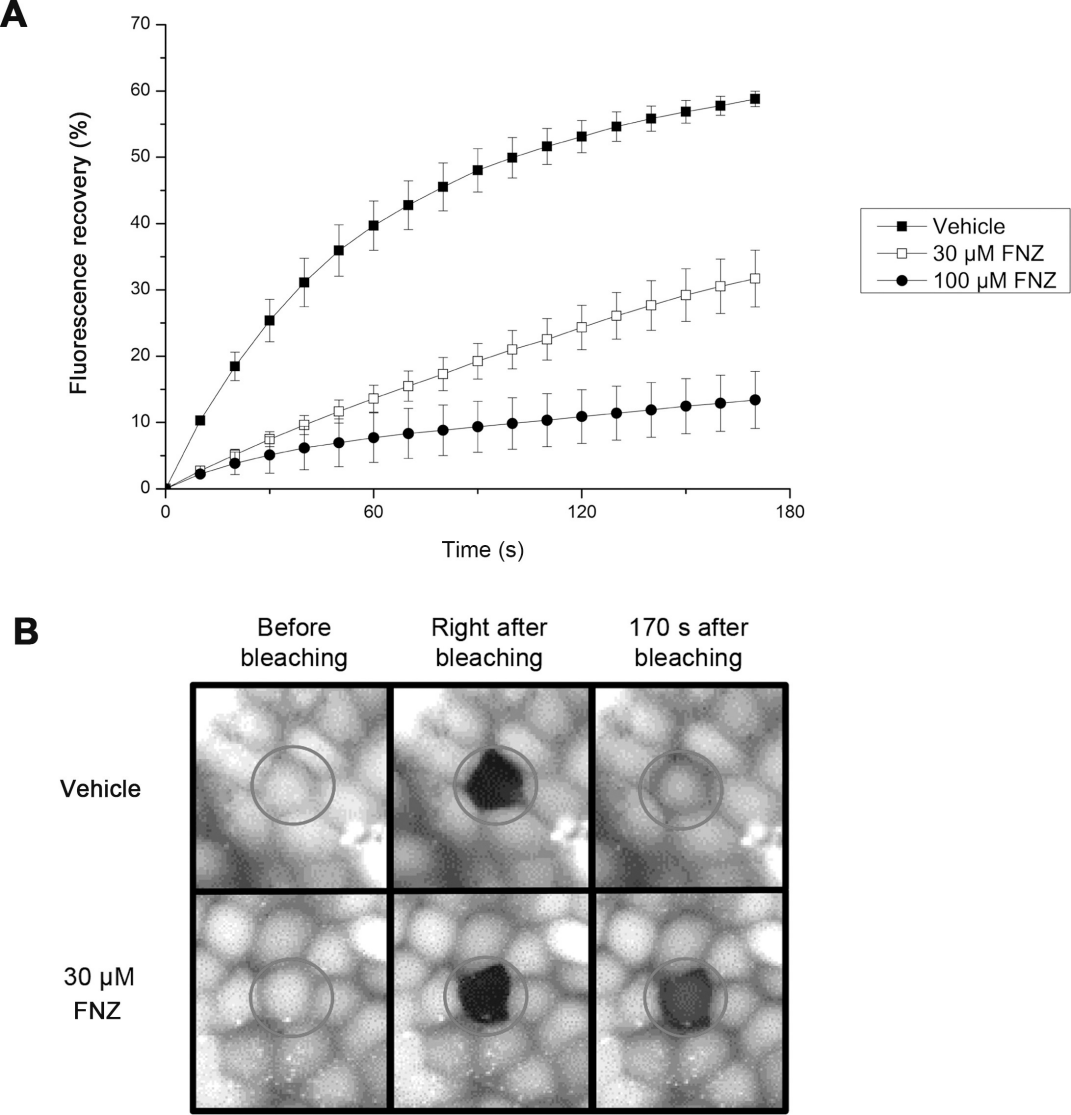
FNZ-induced inhibition of GJIC shown using the gap-FRAP assay. (**A**) Gap-FRAP assay in FRT-Cx43 cells. FRT-Cx43 cells were loaded with calcein-AM and treated with vehicle (n = 4), 30 μM FNZ (n = 9), or 100 μM FNZ (n = 9) for 4 h followed by the gap-FRAP assay. The percentage of fluorescence recovery after photobleaching was plotted against time. Data are presented as the mean ± SD. (**B**) Representative images of the gap-FRAP assay. The images were taken prior to, immediately after, and 170 s after bleaching.

GJIC inhibition by FNZ was also observed using the gap-FRAP assay, a commonly used GJIC assay, in FRT-Cx43 cells. The percentage of fluorescence recovery after bleaching decreased in cells treated with 30 μM FNZ (31.7 ± 4.3%) or 100 μM FNZ (13.4 ± 4.3%) for 4 h compared with cells treated with the vehicle (58.8 ± 0.9%) for 4 h (Fig 2A). Representative images of the gap-FRAP assay are presented in Fig 2B.

### Time course and dose-response relationship of FNZ-induced GJIC inhibition

**Fig 3:**
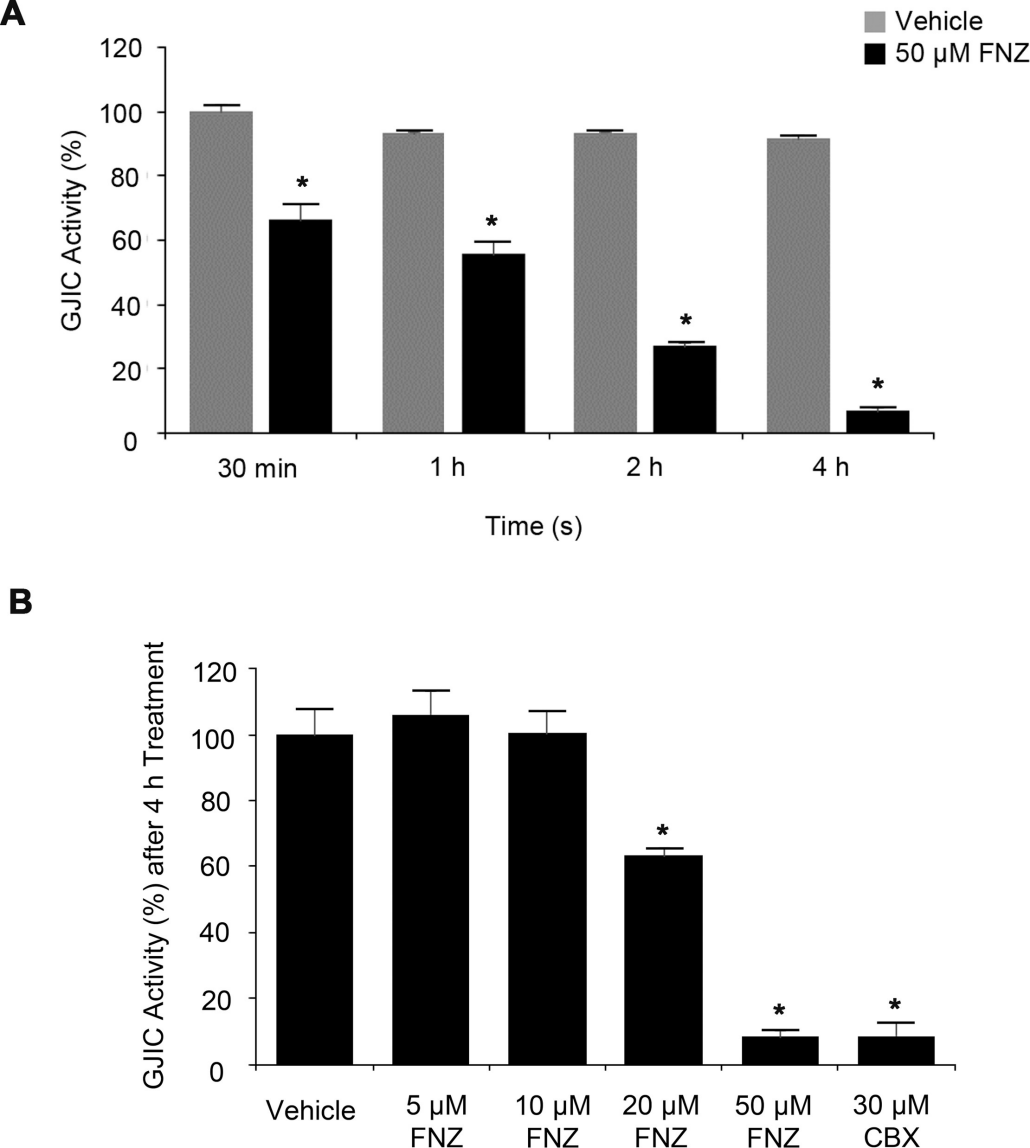
Time course and dose-response relationship of FNZ-induced GJIC inhibition. (**A**) Time course of GJIC inhibition by FNZ. A 1:4 mixture of LN215-YFP^QL^ and LN215-SLC26A4 cells was treated with vehicle or 50 μM FNZ for 30 min, 1 h, 2 h, or 4 h prior to the I-YFP GJIC assay. The percentage of GJIC activity (calculated according to Eq 1 and Eq 2 of the FNZ-treated groups was normalized to that of the group treated with vehicle for 30 min. Data are presented as the mean ± SD (n = 6). (**B**) Dose-response relationship of GJIC inhibition by FNZ. The I-YFP GJIC assays were conducted after 4-h treatment with vehicle; 5, 10, 20, or 50 μM FNZ; or after 10-min treatment with 30 μM CBX. The percentage of GJIC activity was normalized to that of the vehicle-treated group. Data are presented as the mean ± SD (n = 4). * denotes p < 0.05 versus the vehicle-treated group.

To elucidate the underlying mechanism of GJIC inhibition by FNZ, we first attempted to determine the appropriate treatment conditions in terms of duration and concentration. As shown in Fig 3A, FNZ treatment reduced GJIC activities in a time-dependent manner (66.3 ± 5.1% at 30 min, 55.7 ± 3.7% at 1 h, 26.9 ± 1.1% at 2 h, and 6.7 ± 0.8% at 4 h) compared with the group treated with vehicle for 30 min. Next, I-YFP GJIC assays were conducted following treatment with various concentrations of FNZ for the same time (4 h). As shown in Fig 3B, FNZ inhibited GJIC in a dose-dependent manner (5 μM, 106.0 ± 7.2%; 10 μM, 100.5 ± 6.9%; 20 μM, 63.3 ± 2.2%; and 50 μM, 8.4 ± 1.7%). Collectively, we concluded that treating LN215 cells with 50 μM FNZ for 4 h constitutes a potent condition for GJIC inhibition; thus, most subsequent experiments were conducted under this condition.

### Slow reversal of FNZ-induced GJIC inhibition

**Fig 4:**
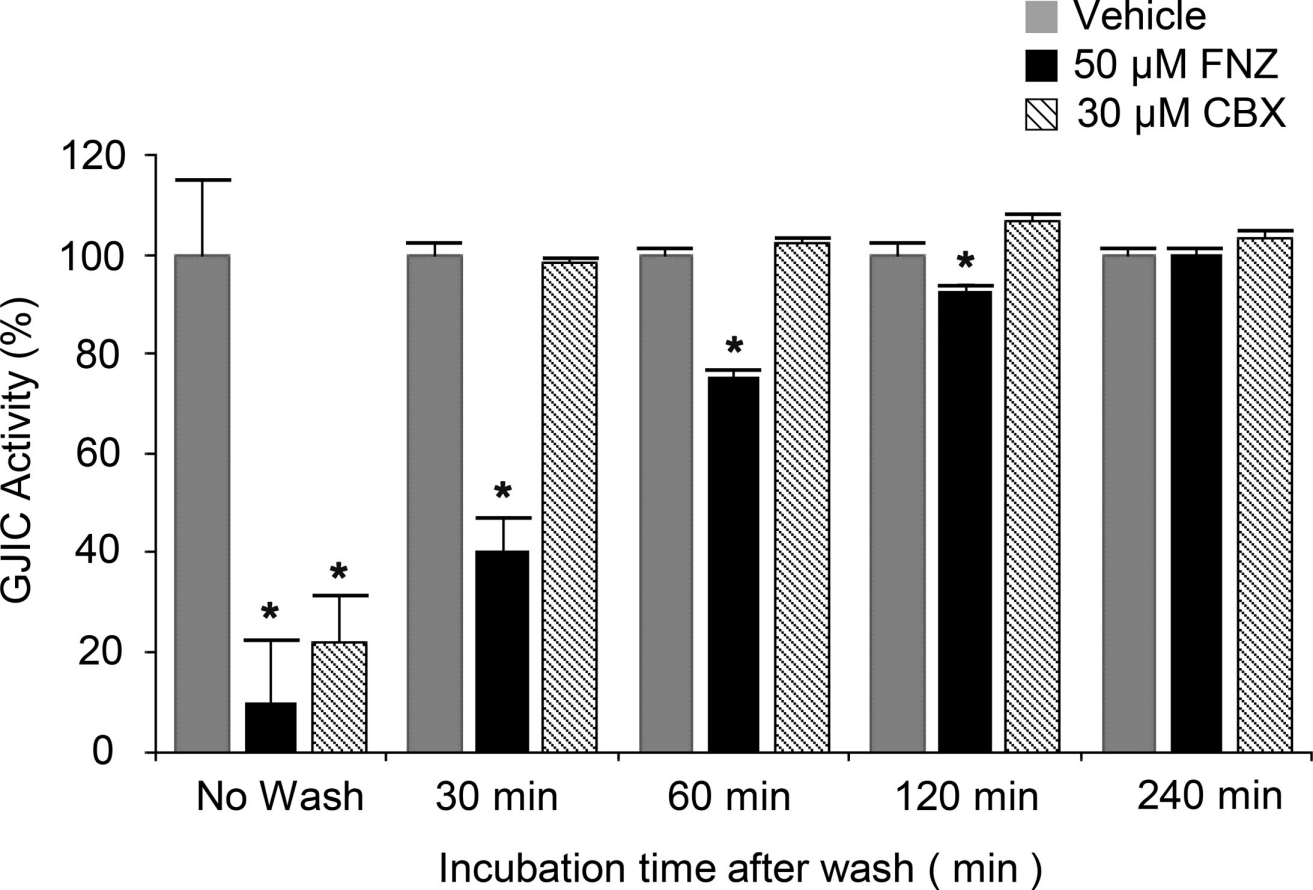
Reversibility of FNZ-induced GJIC inhibition. A 1:4 mixture of LN215-YFP^QL^ and LN215-SLC26A4 cells was treated with vehicle or 50 μM FNZ for 4 h or 30 μM CBX for 10 min. Next, the cells were rinsed twice with C-solution and further incubated in the growth medium without any chemicals for 30, 60, 120, or 240 min prior to the I-YFP GJIC assay. The percentage of GJIC activity of the FNZ or CBX- treated groups was normalized to that of the vehicle-treated groups. The bars in the graph represent the mean ± SD (n = 8). * denotes p < 0.05 versus the vehicle-treated group.

To examine how rapidly FNZ-mediated GJIC inhibition is reversed following FNZ removal, I-YFP GJIC assays were conducted in LN215 cells treated with vehicle or 50 μM FNZ for 4 h, or 30 μM CBX for 10 min, and then washed. GJIC activities were measured prior to washing or 30, 60, 120, or 240 min after washing. As shown in Fig 4, GJIC activities were slowly restored in the FNZ-treated group (10.1 ± 12.4% for no wash, 40.2 ± 6.8% at 30 min, 75.4 ± 1.3% at 60 min, 92.7 ± 1.4% at 120 min, and 99.9 ± 1.2% at 240 min after washing) compared with the CBX-treated group (21.7 ± 9.5% for no wash, 98.3 ± 1.2% at 30 min, 102.4 ± 0.9% at 60 min, 106.5 ± 1.5% at 120 min, and 103.1 ± 1.5% at 240 min after washing).

### Effect of FNZ treatment on cell surface expression of Cx43

**Fig 5:**
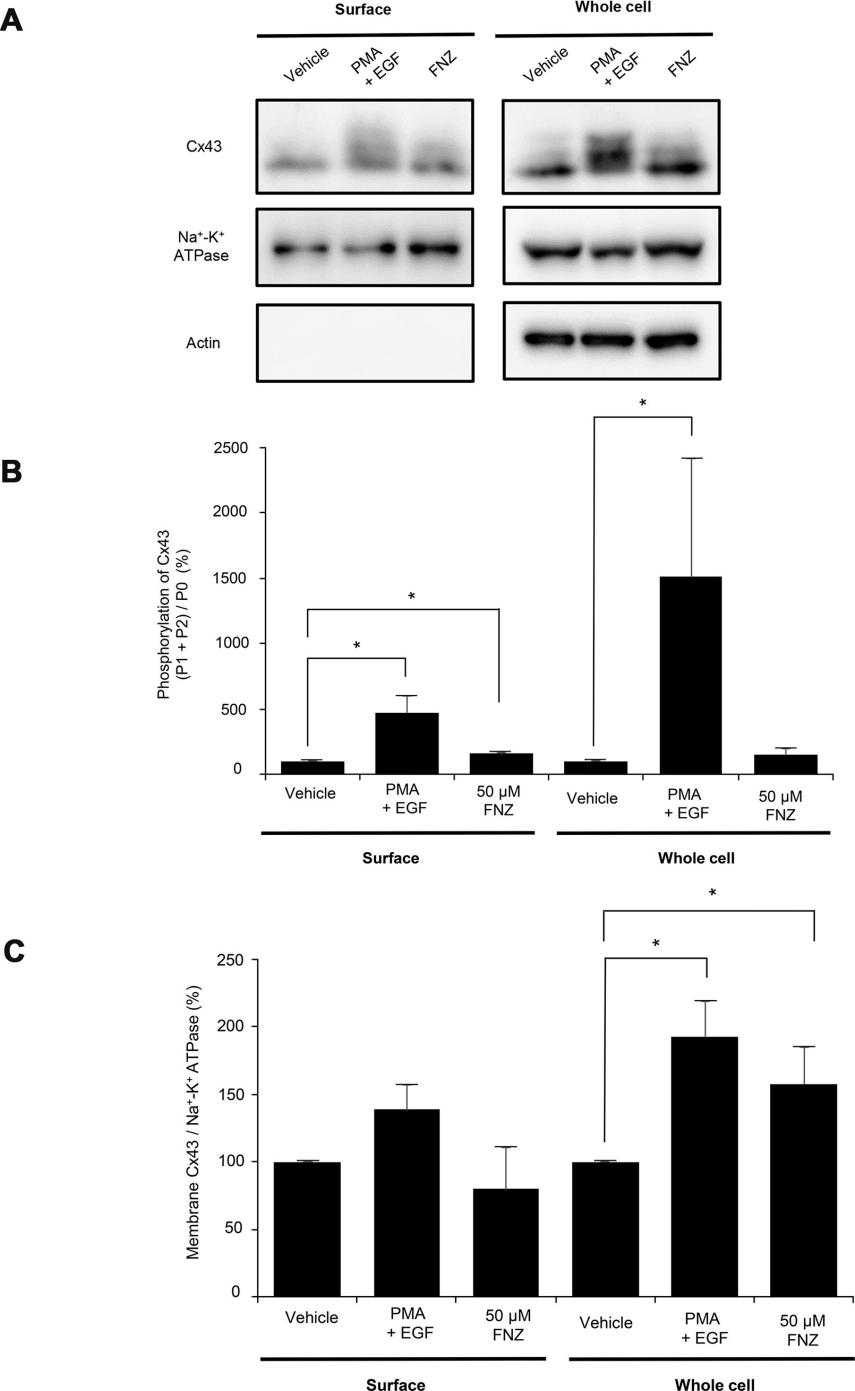
Upregulation of Cx43 phosphorylation without alteration in the total amount of Cx43 on the plasma membrane following FNZ treatment. LN215 cells were treated with vehicle or 50 μM FNZ for 4 h or 100 ng/mL PMA combined with 50 ng/mL EGF for 30 min, followed by in situ biotinylation. Whole cell lysates were collected after surface biotinylation. NeutrAvidin agarose resins were incubated with 1 mg of protein to collect the cell surface proteins. The cell surface proteins and whole cell lysates were analyzed by immunoblots with anti-Cx43, anti-Na^+^-K^+^ ATPase, and anti-actin antibodies. (**A**) Representative immunoblot images. The Cx43 and Na^+^-K^+^-ATPase immunoblots were obtained from the lower and upper parts of the same membrane, respectively. The actin immunoblot image used the same samples, but a separate membrane. The results of the cell surface protein (left) and whole cell lysate (right) immunoblots are presented. Relative phosphorylation (**B**) and relative total amount (**C**) of Cx43 were calculated as described in the Methods section and presented as bar graphs. The error bars represent SD (n = 3). *, p < 0.05.

Cx43 is the major connexin in LN215 human glioma cells [25]. As only connexins located on the cell surface, not in the cytosol, form GJs, Cx43 expression on the plasma membrane, which is more relevant to GJ activity, was analyzed by cell surface biotinylation followed by immunoblotting using an anti-Cx43 antibody. Whole cell lysates were also analyzed by immunoblotting. An anti-Na^+^-K^+^ ATPase antibody was used as the loading control and an anti-actin antibody was used to check for cytoplasmic protein contamination in the biotinylated samples. Representative immunoblot images are presented in Fig 5A (uncropped images are presented in S2 Fig). An increase in the intensity of a slow-migrating band, which has been reported as phosphorylated Cx43, was observed in phorbol-12-myristate-13-acetate and epidermal growth factor (PMA+EGF) treated cells (positive control; Fig 5B). Treatment with 50 μM FNZ for 4 h also significantly increased the intensity of slow-migrating bands in the biotinylated samples (156.6 ± 9.2%; Fig 5B), indicating that FNZ increases Cx43 phosphorylation on the cell surface. No significant changes in total Cx43 levels were observed on the cell surface following FNZ treatment (79.5 ± 18.7%; Fig 5C).

### Protein kinase C (PKC) is not involved in GJIC inhibition by FNZ

**Fig 6:**
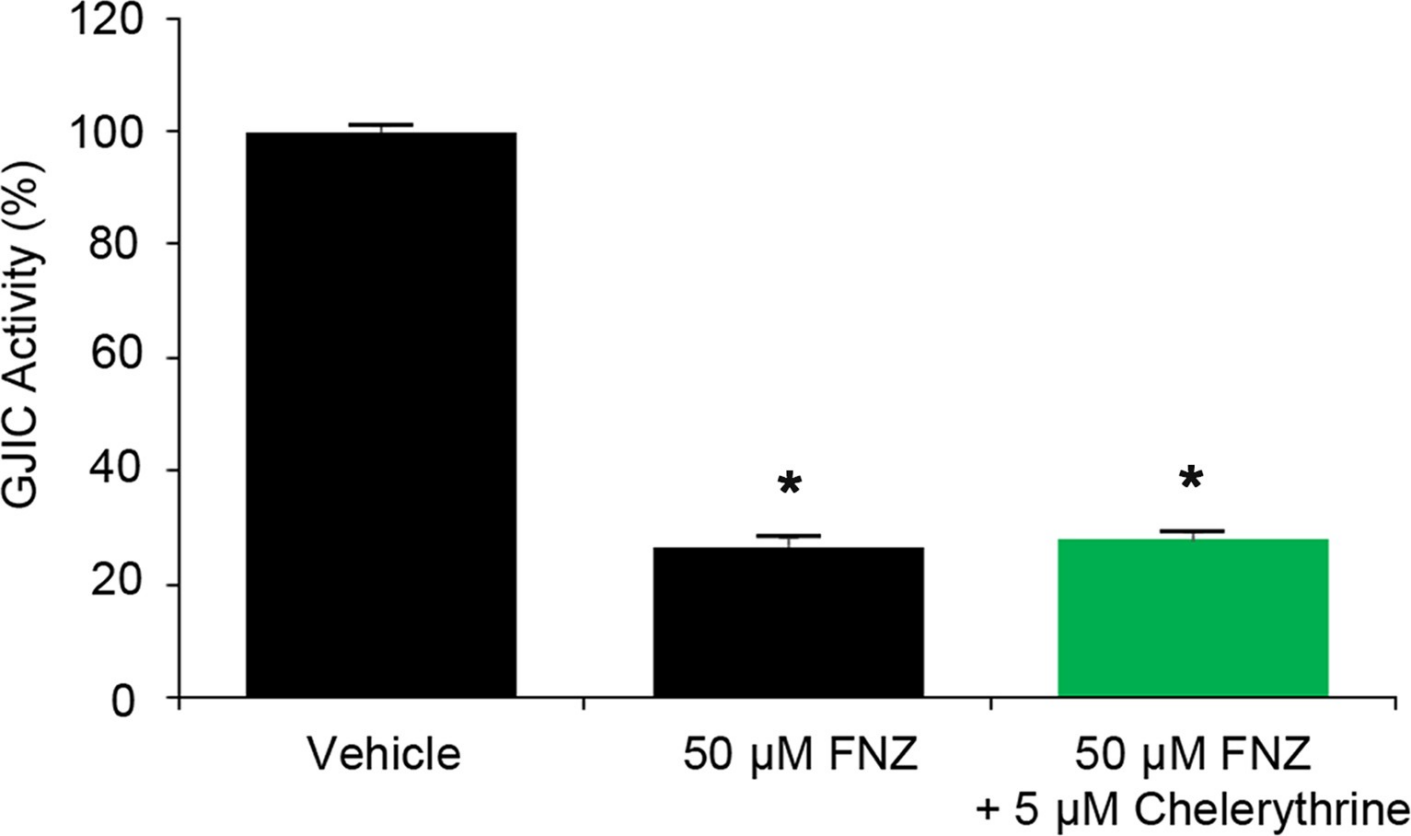
Effect of chelerythrine on FNZ-induced GJIC inhibition. A 1:4 mixture of LN215-YFP^QL^ and LN215-SLC26A4 cells was treated with vehicle, 50 μM FNZ, or 50 μM FNZ together with 5 μM chelerythrine for 4 h prior to the I-YFP GJIC assay. The % of GJIC activity at 20 s of each group was normalized to that of the vehicle-treated group at 20 s. Data are presented as the mean ± SD (n = 4). * denotes p < 0.05 versus the vehicle-treated group.

To investigate whether PKC is involved in GJIC inhibition by FNZ, I-YFP GJIC assays were conducted with the pan-PKC inhibitor, chelerythrine. A 1:4 mixture of LN215-YFP^QL^ and LN215-SLC26A4 cells were treated with vehicle, 50 μM FNZ, or 50 μM FNZ together with 5 μM chelerythrine for 4 h. Chelerythrine did not antagonize FNZ-induced GJIC inhibition (50 μM FNZ, 26.6 ± 1.7%; 50 μM FNZ plus 5 μM chelerythrine, 27.9 ± 1.2%; Fig 6).

### Effects of drugs with pharmacological properties similar to FNZ on GJIC

FNZ is a class IV calcium antagonist [19] with other moderate actions including dopamine D_2_ [20], histamine H_1_ [21], and 5-HT [22] receptor blocking activities. To investigate whether these pharmacological actions of FNZ are related to GJIC inhibition, several drugs, including calcium channel blockers, D_2_ antagonists, H_1_ blockers, and 5-HT antagonists, were used to treat LN215 cells at a concentration of 50 μM for 4 h prior to conducting I-YFP GJIC assays.

**Fig 7:**
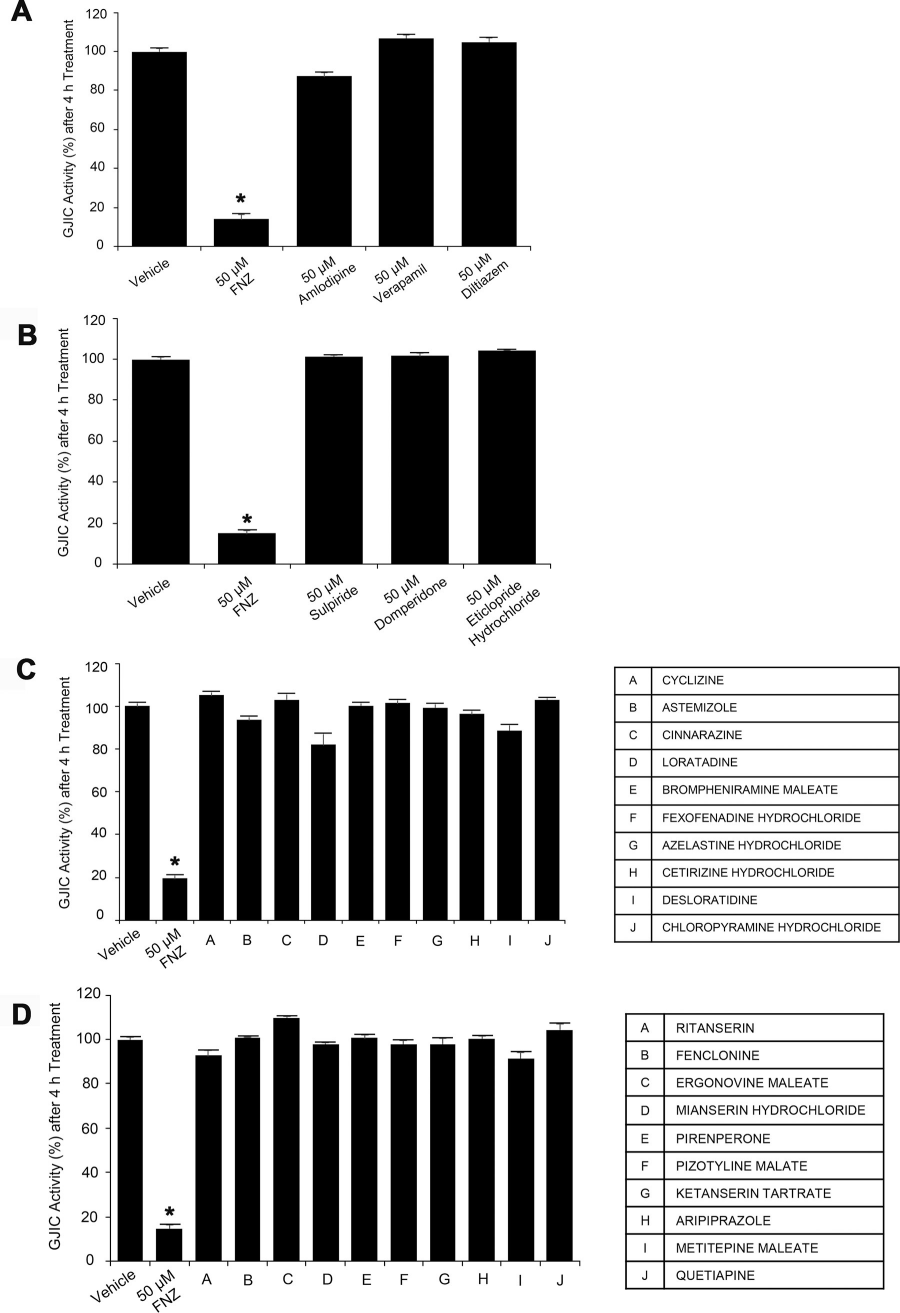
Effect of drugs exhibiting pharmacological actions similar to FNZ on GJIC. The I-YFP GJIC assay was conducted using a 1:4 mixture of LN215-YFP^QL^ and LN215-SLC26A4 cells treated with vehicle, 50 μM FNZ, 50 μM calcium channel blockers (**A**), D_2_ antagonists (**B**), H_1_ blockers (**C**), or 5-HT antagonists (**D**) for 4 h. The drugs used to treat the cells are detailed in the key. The percentage of GJIC activity was normalized to that of the vehicle-treated groups (n = 3). * denotes p < 0.05 versus the vehicle-treated group.

Three calcium channel blockers, amlodipine (87.4 ± 1.7%), verapamil (106.8 ± 1.5%), and diltiazem (104.9 ± 2.0%), did not block GJIC (Fig 7A) under the same treatment conditions as FNZ (14.2 ± 2.4%). None of the D_2_ antagonists (sulpiride, 101.3 ± 0.9%; domperidone, 101.6 ± 1.3%; and eticlopride hydrochloride, 104.2 ± 0.2%) significantly inhibited GJIC (Fig 7B). Similarly, no potent FNZ-like inhibition of GJIC was observed in cells treated with the ten H_1_ blockers or ten 5-HT antagonists (Fig 7C and 7D).

**Fig 8:**
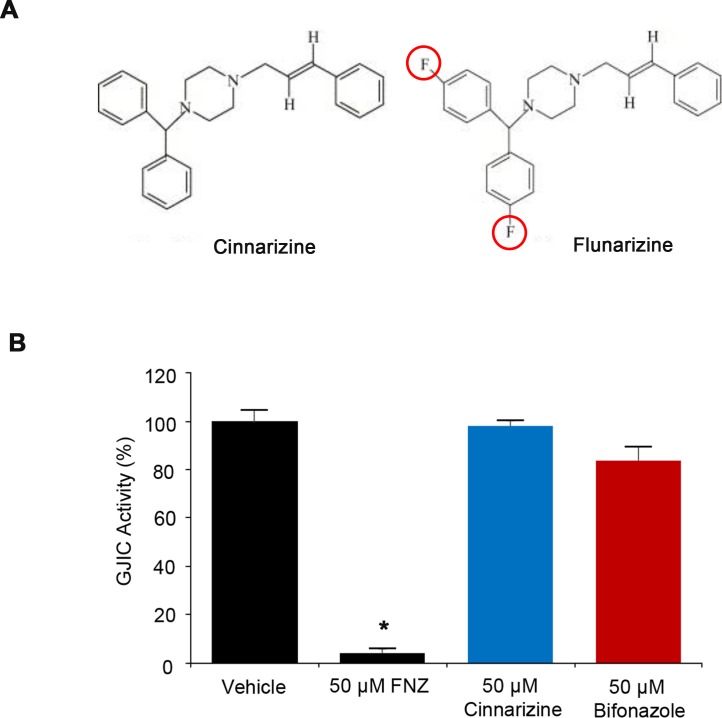
Effect of cinnarizine and bifonazole on GJIC. (**A**) Structural difference between cinnarizine and FNZ. (**B**) I-YFP GJIC assays were conducted using a 1:4 mixture of LN215-YFP^QL^ and LN215-SLC26A4 cells treated with 50 μM FNZ, 50 μM cinnarizine, or 50 μM bifonazole for 4 h. The % of GJIC activity at 20 s of each group was normalized to that of vehicle-treated group at 20 s. Data are presented as the mean ± SD (n = 4). * denotes p < 0.05 versus the vehicle-treated group.

Cinnarizine is a drug with a pharmacodynamic profile similar to FNZ [19], lacking only two fluorine atoms present on the piperazine of FNZ (Fig 8A). In contrast to FNZ (4.2 ± 1.5%), cinnarizine (98.0 ± 2.5%) did not inhibit GJIC under the same treatment conditions as FNZ (Fig 8B). FNZ has also been shown to bind and inhibit calmodulin. To examine whether calmodulin inhibition by FNZ mediated GJIC inhibition in LN215 cells, the effect of bifonazole, a calmodulin antagonist, on GJIC was measured. Treatment with 50 μM bifonazole for 4 h did not inhibit GJIC (83.7 ± 5.7%; Fig 8B).

### FNZ-mediated GJIC inhibition is not attenuated by dopamine, histamine, or 5-HT

**Fig 9:**
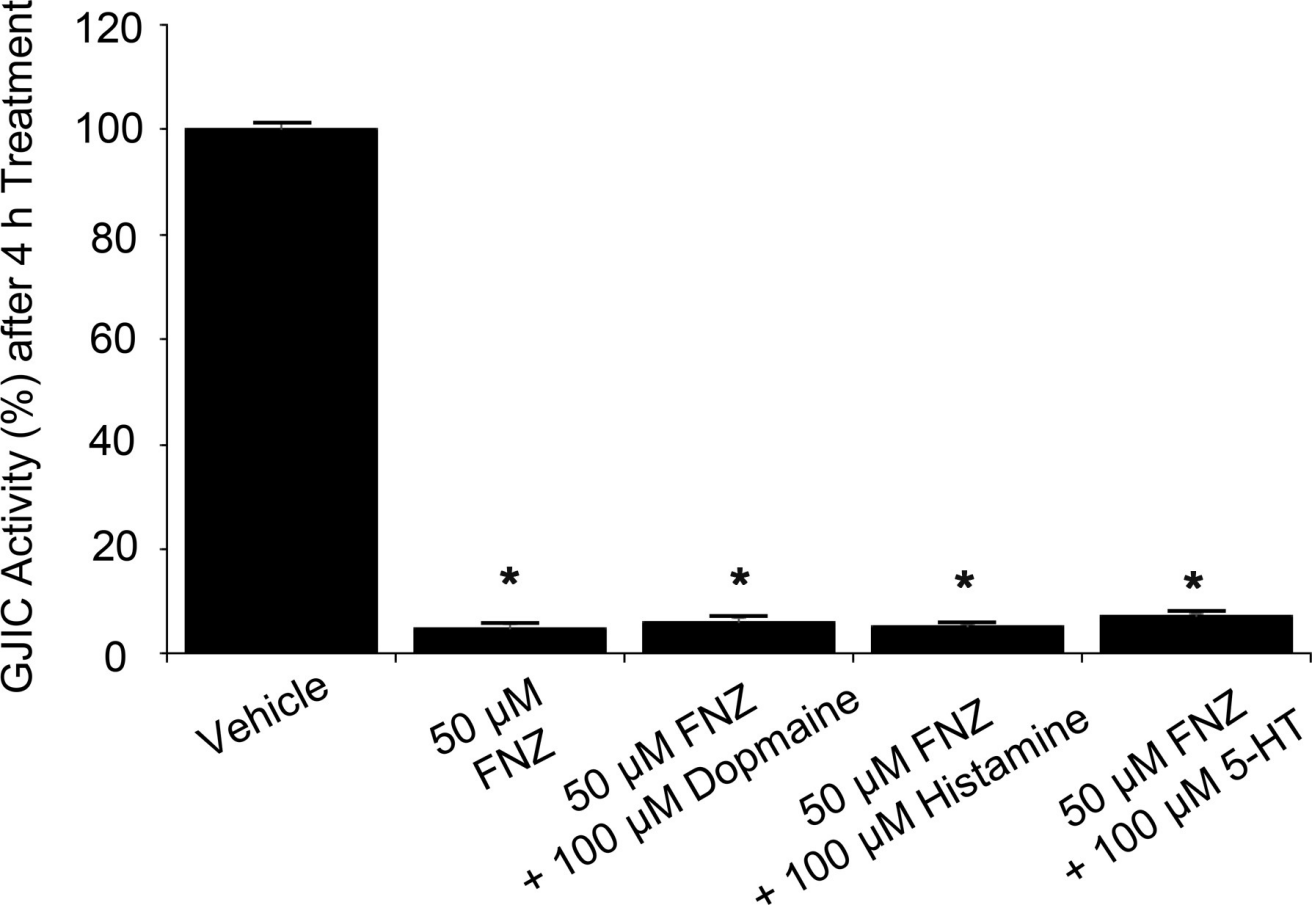
Influence of dopamine, histamine, and 5-HT on FNZ-mediated GJIC inhibition. A 1:4 mixture of LN215-YFP^QL^ and LN215-SLC26A4 cells was treated with vehicle, 50 μM FNZ alone, or 50 μM FNZ combined with 100 μM dopamine, histamine, or 5-HT prior to the I-YFP GJIC assay. The % of GJIC activity was normalized to that of the vehicle-treated. Data are presented as the mean ± SD (n = 6). * denotes p < 0.05 versus vehicle-treated group.

Next, we examined whether dopamine, histamine, or 5-HT are involved in FNZ-induced GJIC inhibition. I-YFP GJIC assays were conducted in cells incubated with 50 μM FNZ alone, or together with 100 μM dopamine, 100 μM histamine, or 100 μM 5-HT for 4 h. FNZ treatment together with dopamine (6.1 ± 0.3%), histamine (5.4 ± 0.2%), or 5-HT (7.1 ± 0.2%) did not interfere with FNZ-induced GJIC inhibition and showed GJIC activities similar to cells treated with FNZ alone (4.9 ± 0.5%; Fig 9). These results suggest that GJIC inhibition by FNZ is not associated with its pharmacological effects on dopaminergic D_2_, histaminergic H_1_, or 5-HT receptors.

### Effect of FNZ on [Ca^2+^]_in_

**Fig 10:**
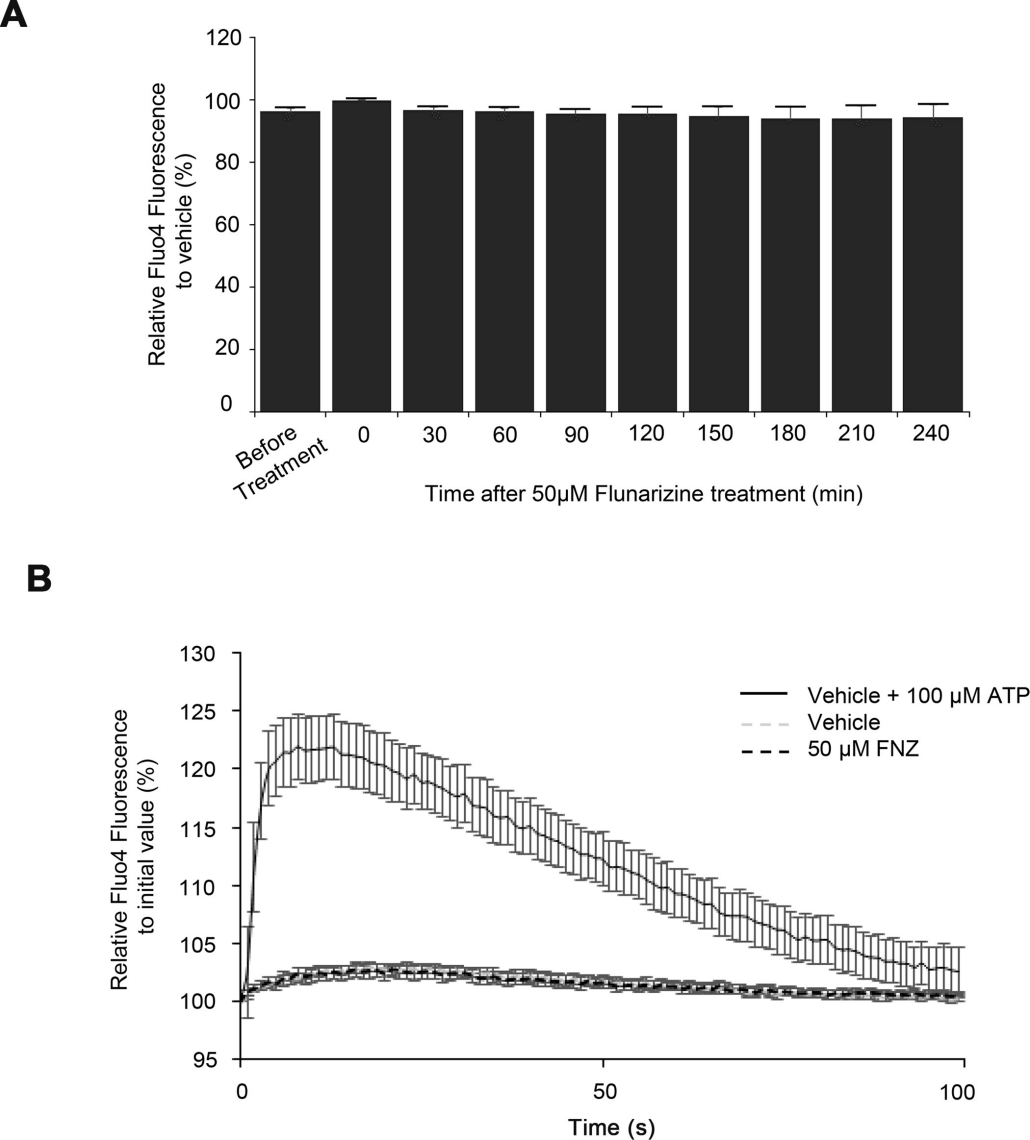
FNZ treatment does not cause a significant change in [Ca^2+^]_in_. (**A**) Observation of [Ca^2+^]_in_ over 4 h. LN215 cells were plated on 96-well plates and grown to full confluence before conducting the Fluo4 calcium assay, as described in the Methods section. Fluo4 fluorescence was measured prior to and following vehicle or 50 μM FNZ treatment at 30 min intervals for 4 h. The fluorescence of the FNZ-treated cells relative to the vehicle-treated cells was calculated at each time point. The error bars represent the SD (n = 3). (**B**) Short-term effect of FNZ on [Ca^2+^]_in_. LN215 cells pre-loaded with Fluo4 dyes were treated with vehicle, 50 μM FNZ, or vehicle together with 100 μM ATP via an automated injector. Fluo4 fluorescence were measured every 0.5 s for 100 s after injection and plotted against time. The vehicle and FNZ traces overlap. Data are presented as mean ± SD (n = 4).

Elevation of [Ca^2+^]_in_ has been shown to inhibit GJ activity [26]. To investigate whether FNZ increased [Ca^2+^]_in_ under the experimental conditions used in this study, a Fluo4 Ca^2+^ assay was conducted in LN215 cells treated with vehicle or 50 μM FNZ. The Fluo4 fluorescence of the cells was measured at 30-min intervals for 4 h. The fluorescence ratio of 50 μM FNZ-treated cells to vehicle-treated cells at each time point did not significantly change over 4 h (Fig 10A). Rapid changes in [Ca^2+^]_in_ were also measured every 0.5 s for 100 s in LN215 cells treated with vehicle, 50 μM FNZ, or vehicle together with 100 μM ATP (positive control). As shown in Fig 10B, FNZ treatment did not cause a significant increase in [Ca^2+^]_in_, while treatment with vehicle together with 100 μM ATP elicited a strong increase in [Ca^2+^]_in._ These results indicate that FNZ inhibition of GJIC is not mediated by an increase in [Ca^2+^]_in_.

## Discussion

The present study demonstrated GJIC inhibition by FNZ, as well as experimental results related to its mechanism of action. In contrast to connexin modulators, such as mefloquine, flufenamic acid, and perhaps CBX, which directly bind to connexins and exhibit rapid kinetics [27], the onset of FNZ-induced GJIC inhibition and its reversal by washing were slow. These slow FNZ kinetics suggest that GJIC inhibition by FNZ is not due to its direct interaction with GJs, but rather is mediated by indirect mechanisms such as changes in (1) cellular or cell surface expression of connexin, (2) the phosphorylation status of connexin, and (3) cellular Ca^2+^ ion concentrations, which were evaluated in this study.

As only connexins on the cell surface can form GJs, we analyzed cell surface Cx43 in LN215 cells treated with FNZ. FNZ treatment significantly increased phosphorylated Cx43 without altering the total amount of Cx43 on the cell surface, as shown in Fig 5. Trafficking, assembly/disassembly, degradation, and gating of GJ channels are highly associated with phosphorylation events [28];[29]. The effects of connexin phosphorylation on GJIC vary according to the type of connexin kinase. Several kinases are involved in Cx43 phosphorylation. Phosphorylation of Cx43 by protein kinase A [30], AKT [31], and casein kinase 1 [32] results in increased GJIC, whereas Cx43 phosphorylation by PKC [33];[29] and tyrosine kinase Src [34];[35] decrease GJIC. FNZ might induce Cx43 phosphorylation by PKC, Src, or other unknown kinases, thereby downregulating GJIC. PMA, a well-known GJ inhibitor (also known as 12-O-tetradecanoylphorbol-13-acetate [TPA]), activates PKC, which directly phosphorylates S368 sites on Cx43, resulting in decreased GJIC [36]. Interestingly, the onset of PMA-induced GJIC inhibition was as slow as FNZ (S3 Fig), which supports the hypothesis that FNZ-induced GJ inhibition is due to increased phosphorylation of Cx43. However, a pan-PKC inhibitor, chelerythrine, did not antagonize FNZ-induced GJIC inhibition (Fig 6). This result suggests that kinases other than PKC might be involved in upregulation of Cx43 phosphorylation by FNZ.

An increase in [Ca^2+^]_in_ inhibits GJs, possibly via the activation of calmodulin [26]. In contrast, FNZ decreases [Ca^2+^]_in_ [19] and has also been shown to bind and inhibit calmodulin [37], but block GJs. To investigate whether FNZ increases [Ca^2+^]_in_ under the treatment conditions used in this study, resulting in GJIC inhibition, Fluo4 calcium assays were conducted during the FNZ treatment period. FNZ itself did not upregulate [Ca^2+^]_in_, as shown in Fig 10. Next, the effect of bifonazole, a calmodulin antagonist, on GJIC was examined to determine whether calmodulin inhibition by FNZ caused GJIC inhibition in LN215 cells; bifonazole did not inhibit GJIC (Fig 8B). These results suggest that GJIC inhibition by FNZ is not mediated by an alteration in cellular Ca^2+^ concentration or calmodulin inhibition.

FNZ has diverse pharmacological effects; it not only antagonizes the entry of calcium into cells [19], but also inhibits the functions of several neurotransmitter receptors including dopamine D_2_ [38], histamine H_1_ [21], and 5-HT receptors [22]. To examine whether these previously established actions are related to GJIC inhibition by FNZ, we assessed the effects of chemicals that have pharmacological effects similar to FNZ on GJIC. None of the three calcium blockers, three D_2_ antagonists, ten H_1_ antagonists, or ten 5-HT antagonists potently inhibited GJIC in LN215 cells. Cinnarizine, which has a structure and pharmacodynamics profile similar to FNZ, did not inhibit GJIC (Fig 8B). Furthermore, FNZ inhibition of GJIC was not attenuated by co-treatment with a high concentration of dopamine, histamine, or 5-HT together with FNZ. Collectively, these data suggest that FNZ-mediated GJIC inhibition is not associated with its previously established pharmacological actions.

FNZ has been used for migraine prophylaxis and epilepsy adjuvant therapy [19]. Interestingly, GJs are also associated with these two diseases. Initiation of cortical spreading depression (CSD), which triggers migraine auras and pain, is associated with astrocytes [2], in which Cx43 is predominantly expressed, as in LN215 [39]^,^[40]. CSD depends on neuronal-glial communication mediated by GJs [2];[5], which have been suggested as therapeutic targets for migraines. Tonabersat, a neuronal-glial GJ inhibitor, was is a candidate for migraine prevention [41]. The beneficial effect of FNZ on migraines might be at least partially due to inhibition of Cx43, similar to tonabersat; however, this hypothesis needs to be further investigated. Connexin proteins and mRNAs are upregulated in temporal lobe neocortices [42], hippocampi [43];[44], and cortices [4] of human epileptic tissues. Thus, GJ inhibitors have been suggested as potential antiepileptic agents [45];[46]. Agents that block GJs, such as carbenoxolone [47], quinine [48];[49], meclofenamic acid [50];[51], and tonabersat [52], have anticonvulsant effects [53]. Thus, the anticonvulsant effects of FNZ might be due, at least in part, to GJ modulation.

## Supporting information

S1 Fig Effect of FNZ on SLC26A4. LN215 cells co-expressing YFP^QL^ and SLC26A4 were plated on 96-well plates and cultured for 24 h. The cells were then treated with vehicle or 50 μM FNZ for 4 h, or 30 μM PDS_inh_^-^C01, a well-known SLC26A4 blocker, for 10 min prior to the I-YFP GJIC assay. The percentage of YFP fluorescence was plotted against time. Data are presented as the mean ± SD (n = 4).
(TIF)

S2 Fig Uncropped immunoblots images from Fig 5. (TIF)

S3 Fig Time course of GJIC inhibition by PMA. I-YFP GJIC assays were conducted using a 1:4 mixture of LN215-YFP^QL^ and LN215-SLC26A4 cells treated with vehicle or 100 ng/mL PMA for 30 min, 1 h, 2 h, or 4 h. The % of GJIC activity of the PMA-treated group was normalized to that of the group treated with vehicle for 30 min. Data are presented as the mean ± SD (n = 4).
(TIF)
