# Supplementary figures and images for: Inhibition of gap junctional intercellular communication by an anti-migraine agent, flunarizine
Source: PLoS One. 2019 Sep 12;14(9):e0222326. doi: 10.1371/journal.pone.0222326 (PMC6742374; doi:10.1371/journal.pone.0222326)

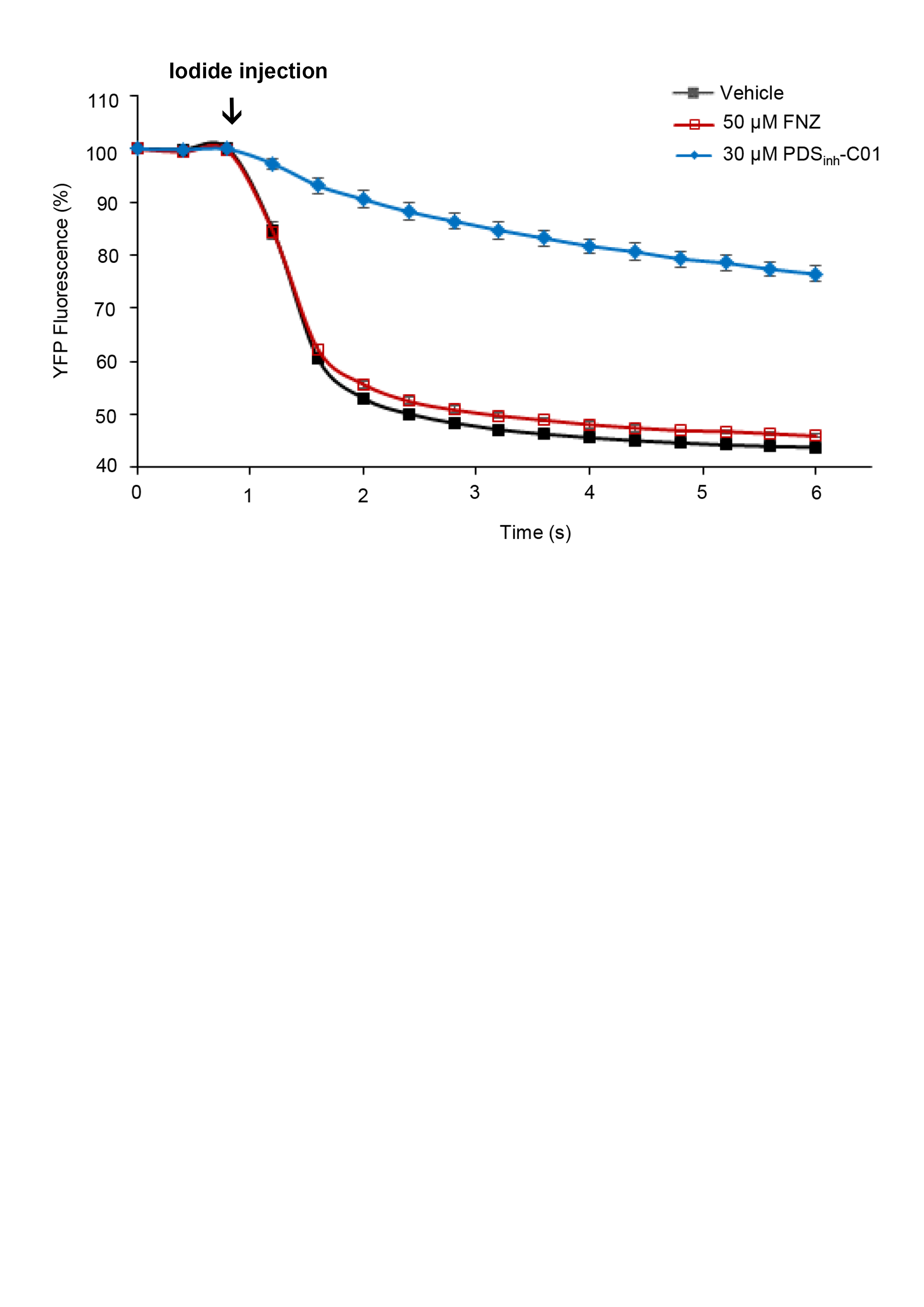

Supplement: S1 Fig — LN215 cells co-expressing YFPQL and SLC26A4 were plated on 96-well plates and cultured for 24 h. The cells were then treated with vehicle or 50 μM FNZ for 4 h, or 30 μM PDSinh-C01, a well-known SLC26A4 blocker, for 10 min prior to the I-YFP GJIC assay. The percentage of YFP fluorescence was plotted against time. Data are presented as the mean ± SD (n = 4). (TIF) [file pone.0222326.s001.tif]

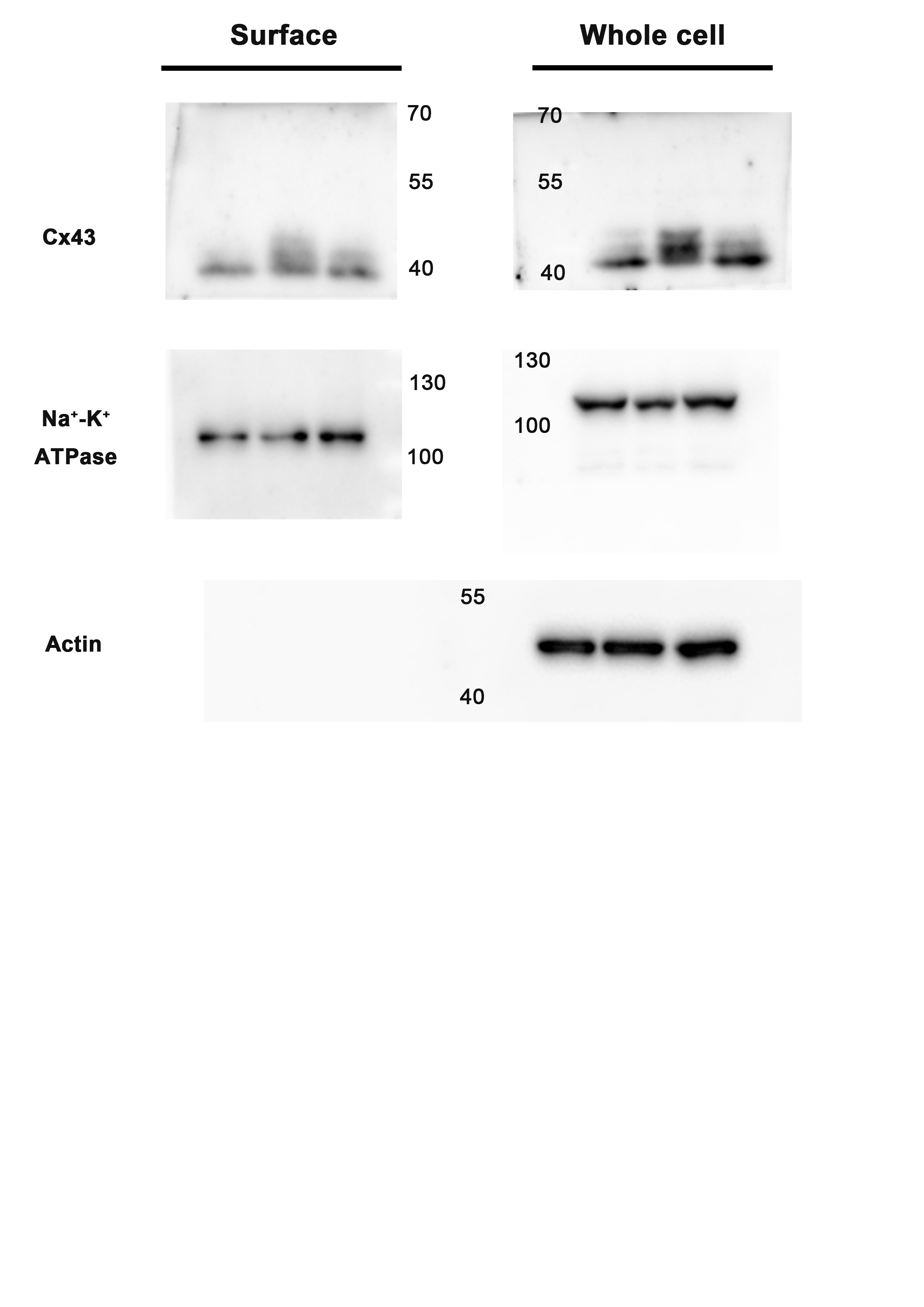

Supplement: S2 Fig — (TIF) [file pone.0222326.s002.tif]

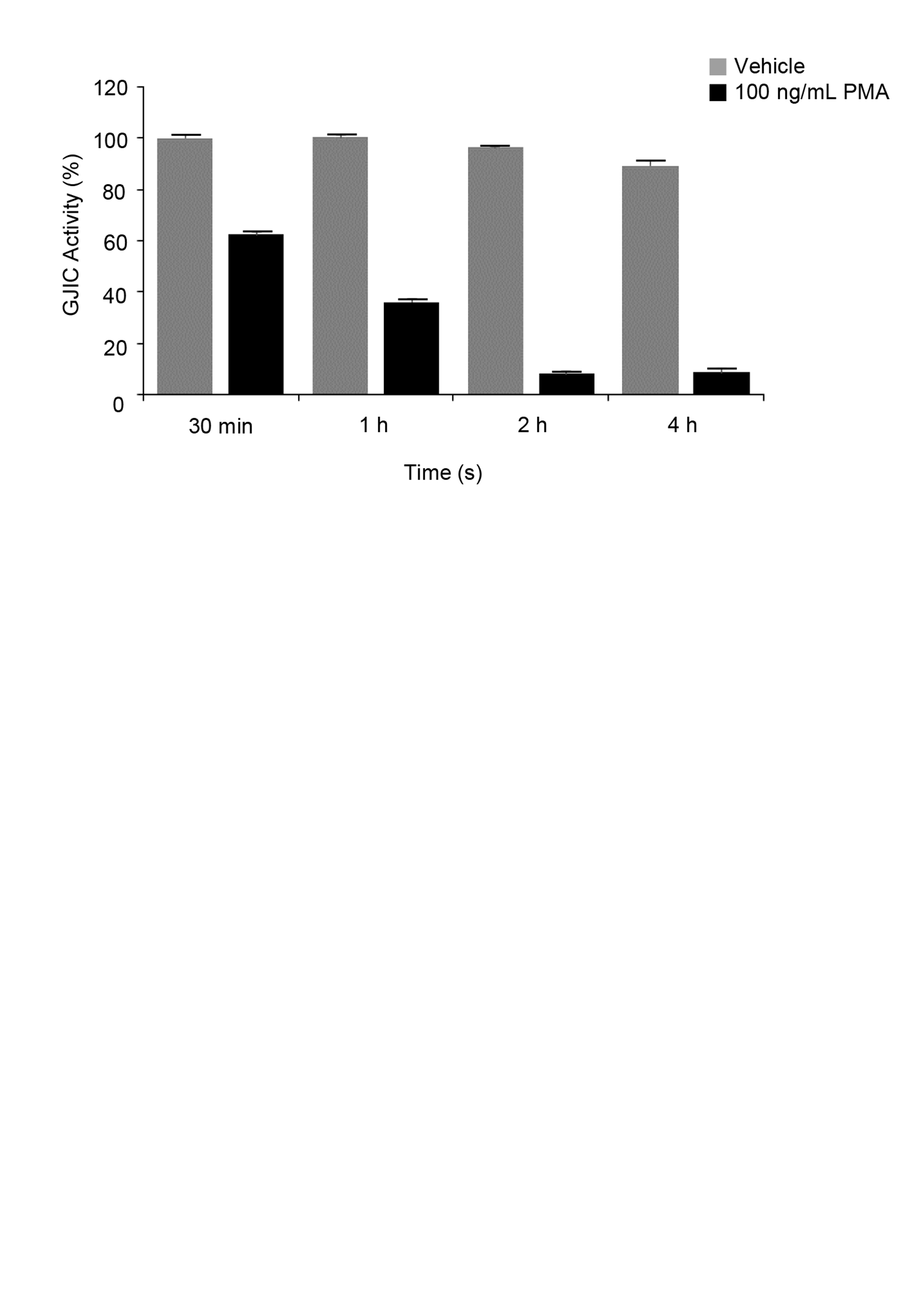

Supplement: S3 Fig — I-YFP GJIC assays were conducted using a 1:4 mixture of LN215-YFPQL and LN215-SLC26A4 cells treated with vehicle or 100 ng/mL PMA for 30 min, 1 h, 2 h, or 4 h. The % of GJIC activity of the PMA-treated group was normalized to that of the group treated with vehicle for 30 min. Data are presented as the mean ± SD (n = 4). (TIF) [file pone.0222326.s003.tif]
